# Combined modality chemoradiation in elderly oesophageal cancer patients

**DOI:** 10.1038/sj.bjc.6603821

**Published:** 2007-05-29

**Authors:** S E Anderson, B D Minsky, M Bains, A Hummer, D Kelsen, D H Ilson

**Affiliations:** 1 Department of Medicine, Memorial Sloan-Kettering Cancer Center, 1275 York Avenue, New York, NY 10021, USA; 2 Department of Radiation Oncology, Memorial Sloan-Kettering Cancer Center, 1275 York Avenue, New York, NY 10021, USA; 3 Department of Thoracic Surgery, Memorial Sloan-Kettering Cancer Center, 1275 York Avenue, New York, NY 10021, USA; 4 Department of Biostatistics, Memorial Sloan-Kettering Cancer Center, 1275 York Avenue, New York, NY 10021, USA

**Keywords:** elderly patients, oesophageal cancer, combined modality therapy

## Abstract

We present a single institution experience with 5-FU, mitomycin-C based chemoradiation for the primary treatment of elderly patients with oesophageal cancer. Twenty-five patients with a median age of 77 years (range 66–88) with a diagnosis of stage II–III squamous cell or adenocarcinoma of the oesophagus were treated at Memorial Sloan Kettering from 1996 to 2001 with two cycles of concurrent 5-FU, mitomycin-C and 50.4 Gy. Owing to age and comorbidity, these patients were not considered surgical candidates. The Charlson comorbidity score was used to evaluate patient comorbidity. Nine patients (36%) experienced grade 3–4 haematologic toxicity. Of the 23 patients evaluable for response, 17 patients (68%) had a negative post-treatment endoscopy and CT scan without evidence of progressive disease. Eleven patients (44%) are alive and 10 (40%) remain without evidence of recurrent or progressive oesophageal cancer at a median follow-up of 35 months. The median overall survival was 35 months and 2-year survival 64%. There was no significant difference in overall survival between Charlson score ⩽2 and those with a score ⩾2 (*P*=0.10). Similar survival was observed for patients with adenocarcinoma or squamous carcinoma. Primary chemoradiation with two cycles of 5-FU, mitomycin-C, and 50.4 Gy in elderly patients is an active regimen with moderate toxicity, despite the advanced age and heavy comorbidity burden of this cohort. Patients with local/regional oesophageal cancer with adequate functional status should not be excluded from potentially curative treatment based on age alone.

The incidence of oesophageal adenocarcinoma has had the most rapid rise of any solid tumour malignancy in the United States (Devesa *et al*, 1998). Over 14 550 Americans were diagnosed with this malignancy in 2006 (Jemal *et al*, 2006). Most patients have stage III or IV disease at presentation. Unfortunately, there has been little change in 5-year survival, which remains less than 10% (Enzinger and Mayer, 2003).

Elderly patients with early stage oesophageal cancer treated with surgery alone appear to achieve similar survival benefit compared to younger patients without increased mortality (Alexiou *et al*, 1998). However, most series indicate that a significantly smaller proportion of elderly patients diagnosed with oesophageal cancer is actually referred for oesophagectomy compared to younger patients (Sabel *et al*, 2002; Bonavina *et al*, 2003), indicating significant patient selection. Few series report patients' coexisting medical conditions (Poon *et al*, 1998, Ma *et al*, 2006). Most series indicate comparable to higher rates of post-operative morbidity, and comparable rates of post-operative mortality in patients over the age of 70 referred for oesophagectomy (Jougon *et al*, 1997; Kinugasa *et al*, 2001). Adenocarcinoma is the dominant histology in Western surgical series in these patients. Several investigations of elderly patients treated with radiation alone indicate no difference in response or survival when compared to younger patients (Hishikawa *et al*, 1991; Yamakawa *et al*, 1994).

The Radiation Therapy Oncology Group (RTOG) trial 85-01 established the superiority of chemotherapy with cisplatin, 5-FU and concurrent radiation over radiation alone (Herskovic *et al*, 1992). Patients randomised to chemoradiation demonstrated improved overall survival (26 *vs* 0% at 3 years) and median survival (12.5 *vs* 9 months) compared to the radiation-alone arm. This regimen has been considered the standard of care for treatment of patients with locally advanced oesophageal cancer treated without surgery. Unfortunately, given the substantial acute toxicity of this regimen it has commonly been reserved for treatment of younger patients with good performance status. Toxicity on the RTOG trial was substantial. Sixty-four percent of patients treated with chemoradiation experienced severe or life-threatening side effects compared to 28% of patients treated with radiation alone. Only 23% of patients enrolled were over age 70.

Little has been reported about the use of primary chemoradiotherapy without surgery in elderly patients with locally advanced oesophageal and squamous cancers. In addition, limited data are available regarding the response or tolerance of combined modality chemoradiation in elderly patients with oesophageal cancer. One recent series indicated comparable post-operative mortality rates even in patients over the age of 70 receiving preoperative combined chemoradiotherapy followed by surgery (Rice *et al*, 2005) compared to elderly patients undergoing surgery alone.

Although the majority of cancers in the United States occur in the elderly, older American are under-represented in clinical trials (Hutchins *et al*, 1999). A study of the Southwest Oncology Group (SWOG) compared the enrollment of older Americans onto SWOG trials from 1993 to 1996 to the actual proportion of older persons with cancer. Although patients over the age of 65 accounted for 63% of cancers in the United States, only 25% of patients enrolled on SWOG trials were over 65 years. A European analysis corroborates the under-representation of elderly patients entered on clinical trials (Monfardini *et al*, 1995). Reasons for the under-representation of elderly patients on clinical trials in oesophageal cancer are unclear. Physician concern regarding comorbidities, the possibility of increased toxicity, and lack of data supporting the efficacy of treatment may be contributing factors.

To determine the tolerability and efficacy of chemoradiation in this population, we reviewed our institutional experience over a 5-year period in persons over 65 years with locally advanced oesophageal cancer treated with 5-FU, mitomycin, and concurrent radiotherapy as primary treatment of oesophageal cancer. We opted to employ the combination of 5-FU and mitomycin with radiation, as small patient series with oesophageal cancer treated with this regimen suggest a more favourable toxicity profile in comparison to conventional 5-FU and cisplatin (Coia *et al*, 1991a, 1991b). We defined an elderly population according to Social Security and Medicare regulations as persons aged 65 years or older.

## MATERIALS AND METHODS

Given the age and comorbidity of this older patient population, definitive chemotherapy and radiation was planned as primary therapy without surgery. Patients 65 years and older diagnosed with locally advanced oesophageal cancer at Memorial Sloan-Kettering Cancer Center between January 1996 and April 2001, who were selected to receive chemoradiation with two cycles of 5-FU and mitomycin as first-line treatment were included in this analysis. Patients were treated in hospital for close monitoring during the two cycles of chemotherapy, as many patients had multiple comorbid conditions. Specifically 7 patients had a history of arrhythmia, 13 had hypertension, 8 patients had documented peripheral vascular and/or coronary artery disease, and 3 patients had a history of heart failure.

### Treatment regimen

Therapy consisted of 5-FU at a dose of 750–1000 mg m^−2^ per day for 4 days given by continuous infusion weeks 1 and 5. Mitomycin was given at a dose of 7–10 mg m^−2^ on days 1 and 29 of chemoradiation.

### Radiation therapy

Radiation therapy was delivered with megavoltage equipment (15 MV) using a multiple field technique. Patients were treated 5 days per week at 1.8 Gy per day. All fields were treated each day, and portal films were obtained of at least two fields per week, or more often if needed. Treatment was delivered with four fields (AP/PA and opposed laterals) such that the dose did not vary by >5% over the entire target volume. The dose was prescribed to the isodose line that covered the volume at risk. Lung inhomogeneity corrections were used.

The superior and inferior borders of the radiation field were 5 cm beyond the primary tumour. The lateral, anterior, and posterior borders of the field were ⩾2 cm beyond the borders of the primary tumour. The tumour size was defined by endoscopic ultrasound (EUS), barium swallow, or CT scan (whichever was larger). The primary and the regional lymph nodes were included.

### Pretreatment evaluation

All patients had an upper endoscopy. Ten patients also had EUS performed at the discretion of their referring gastroenterologists. All patients had CT scans of the chest and abdomen before treatment and 11 patients had PET scans as part of their pretreatment evaluation. Patients without EUS were clinically staged as N1 on the basis of visible lymphadenopathy on CT scan, and T3 on the basis of visible oesophageal mass on CT scan. Post-treatment response was evaluated via endoscopy and CT scan. Post-treatment evaluations were conducted 8–16 weeks after completion of all treatment.

To give a descriptive analysis of this cohort's comorbidity burden, we used the Charlson comorbidity index (Charlson score; Charlson *et al*, 1987). The Charlson score is a widely used comorbidity scale validated to correlate with 1-year mortality risk in cancer patients (Newschaffer *et al*, 1997; Reid *et al*, 2001). Nineteen medical conditions generating a relative increased risk of death are weighted, and calculated as four ordinal categories: 0, 1–2, 3–4, and 5+.

### Statistical analysis

To test if a Charlson score ⩾2 was related to increased toxicity or increased death, the Fisher's exact test was used. Overall survival was based on the Kaplan–Meier method. The log-rank test was used to compare differences in length of survival between groups. The *t*-test was performed to assess if increased age was related to toxicity. All statistical analysis was performed using SAS Software (SAS Institute, Cary, NC, USA).

### Evaluation of response

Achievement of a complete response required a negative endoscopy and biopsy and CT scan without evidence of disease progression or recurrence. Patients were seen in clinic every 3–6 months with follow-up endoscopies and CT scans. Surgery was not planned in this elderly patient population with comorbidities.

## RESULTS

### Patients

Twenty-five patients over age 65 with locally advanced oesophageal cancer were treated during this period. Patient age ranged from 66 to 88 years (median 77 years). Twenty-three patients (92%) were 70 years or older. There were 14 women and 11 men. All patients had a minimum Karnofsky performance status of 70%. Twelve patients had adenocarcinoma and 13 had squamous cell cancer. Twenty-one patients (84%) had clinically staged T3/T4 or N1 disease by EUS or CT scan. Baseline characteristics are listed in Table 1.

The prevalence of comorbidity, defined as a Charlson score ⩾1, was 84%. The mean Charlson score was 2. Seventy-two percent of patients had a Charlson score ⩾2 and 36% percent of patients had a Charlson score ⩾3. Five patients (20%) were diabetic and 10 (40%) had chronic obstructive pulmonary disease. Twelve patients (36%) had a prior or concurrent malignancy. Nine patients (36%) had peripheral vascular or cerebrovascular disease. One patient had a prior myocardial infarction and three patients (12%) had a history of heart failure.

Fifteen (60%) patients were active or former cigarette smokers with a mean pack/year history of 18 years (range 10–100). Twenty-one patients (84%) presented with dysphagia. Weight loss was also notable in most patients with 15 (60%) experiencing a mean 10-pound decrease from baseline (range 5–30 pounds). Three patients (15%) received feeding tubes for nutritional support before initiation of treatment.

### Treatment delivery

Twenty-two of 25 patients completed all planned treatment. For cycle 1 of treatment patients received mitomycin 7–10 mg m^−2^ on day 1 (3 patients 10 mg m^−2^, 19 patients 8 mg m^−2^, and 3 patients 7 mg m^−2^). A total of 750–1000 mg m^−2^ 5-FU was given in days 1–4 (18 patients 750 mg m^−2^ and 7 patients 1000 mg m^−2^). Although no planned dose selection was made in this retrospective series, older patients were generally treated with lower chemotherapy doses.

For cycle 2, three patients (15%) required dose attenuation. The remainder of patients received the same dose as in cycle 1. One patient had a 33% dose reduction of 5-FU from 750 to 500 mg m^−2^ due to excess gastrointestinal toxicity. Two patients required omission of mitomycin from cycle 2 secondary to haematologic and gastrointestinal toxicity, respectively.

### Toxicity

All patients were evaluable for toxicity. Nine patients (36%) required admission for toxicity management. Four patients required admission after the first week of chemoradiation for toxicity management (two neutropenic fever, one dehydration, one heart failure management.) Five patients required admission for toxicity management after the second course of chemoradiation (two oesophagitis, one cellulitis, one radiation pneumonitis, one neutropenic fever.) Nine patients (36%) required treatment delay. All patients completed all planned radiotherapy. No patient required a feeding tube during chemoradiation treatment.

Haematologic toxicity was moderate; mean haematologic nadirs are provided in Table 2. Nine patients (36%) experienced grade 3 or 4 haematologic toxicity: neutropaenia 7 (six grade 4, one grade 3); thrombocytopaenia 2 (grade 4). No death related to treatment toxicity was observed. Toxicity data are recorded in Table 2.

### Treatment outcome and survival

Twenty-five patients completed chemoradiation and 23 patients are evaluable for treatment response. Two patients were not evaluable for response. One patient died 32 days after completion of treatment secondary to heart failure related complications. A second patient died 37 days after last treatment from complications related to ischemic heart disease. Although the post-treatment CT scan was suspicious for disease progression, this patient did not undergo post-treatment endoscopy.

Twenty-three patients had post-treatment CT scans and endoscopies. Seventeen patients (68%) had no evidence of persistent cancer on endoscopy and a CT scan without evidence of disease progression. Three patients (15%) had evidence of a major response at endoscopy, but had local persistence of their tumours. Two of these patients underwent salvage therapy with one patient achieving a complete response. The patient who achieved a complete response to salvage treatment underwent two applications of high-dose rate intraluminal brachytherapy to the lower oesophagus for a total dose of 1000 cGy. The other patient who underwent salvage received two treatments of photodynamic therapy. Unfortunately, she experienced worsening symptoms of dysphagia and ultimately died of metastatic disease shortly thereafter. The third patient underwent surgical resection for residual carcinoma *in situ* and died from post-operative complications. The remaining three patients' (15%) post-treatment evaluations revealed persistent or progressive disease either on CT or endoscopy.

**Figure 1:**
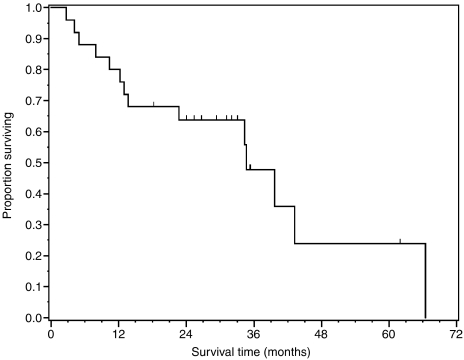
Overall survival.

**Figure 2:**
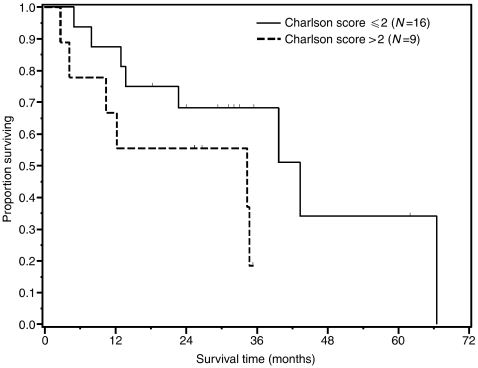
Overall survival by Charlson score.

Eleven patients are alive (44%) and 10 (40%) remain without evidence of disease with a median follow-up of 32 months (range 18–62). Median overall survival for the entire cohort is 35 months (95% CI, 14–66). One- and 2-year overall survival is 80% (95% CI, 64–96%) and 64% (95% CI, 45–83%), respectively. Median follow-up for the entire group is 35 months (range 3–66). Survival outcome was similar for patients with adenocarcinoma and squamous carcinoma: of 14 patients who survived and remained free for at least 2 years out from treatment, 6 had adenocarcinoma (50% of 12 patients initially treated) and 8 had squamous carcinoma (62% of 13 patients initially treated). Of seven patients 80 years or older at the start of therapy, three patients (43%) were alive and free of disease for at least 2 years out from treatment (two patients with adenocarcinoma and one with squamous cell carcinoma). There was no significant difference in overall survival between patients with a Charlson score ⩽2 (median 43 months, 95% CI, 23–66) compared to those with a score ⩾2 (median 34 months, 95% CI, 10 not reached), *P*=0.10. There was a correlation between Charlson score ⩾2 and grade 3 or 4 toxicity (*P*=0.03). However, there was no correlation between Charlson score ⩾2 and hospital admission (*P*=0.14). In addition, increased age did not correlate to an increased risk of death (*P*=0.46). Survival curves are shown in Figures 1 and 2.

## DISCUSSION

The National Cancer Database of the American College of Surgeons, reveals that the mean age of patients with oesophageal cancer is 67.3 years (Daly *et al*, 2000). Most patients present with stage III or IV disease. Chemoradiation is the standard non-surgical treatment for patients with locally advanced oesophageal cancer (Coia *et al*, 2000). Almost 50% of patients treated with standard therapy with continuous infusion 5-FU, cisplatin, and radiation in the RTOG 85–01 trial experienced severe or life-threatening haematologic toxicity raising the question regarding tolerance of therapy in an elderly population with oesophageal cancer.

Our retrospective data suggest that chemoradiation with 5-FU and mitomycin in geriatric oesophageal cancer patients is both tolerable and efficacious. Although patient selection is clearly an issue in a retrospectively treated series of patients, nonetheless our patients had a median age of 77 years and 84% presented with at least one comorbid condition. The majority of patients were not surgical candidates due to advanced age and medical comorbidities despite locally advanced disease. Treatment was tolerable with moderate toxicity observed. Grade 4 toxicity was observed in only four patients (16%) and there were no treatment-related deaths. All 25 patients completed treatment and only 3 patients (12%) required dose adjustment. With a 1-year overall survival rate of 80%, 2-year overall survival of 64%, 40% of patients remain alive without evidence of disease. Of note, of the 15 patients treated with 7 or 8 mg m^−2^ mitomycin and 750 mg m^−2^ per day 5-FU, there was no grade 4 toxicity observed and only two patients (8%) required admission for toxicity management. Two-year survivorship was comparable for adenocarcinoma and squamous cell carcinoma, and patients 80 years and older also achieved a similar 2-year survival. Survival results in this series meet or exceed the survival reported in the original RTOG 85–01 trial, again likely a function of patient selection and also of potentially superior staging of patients in this series, using EUS, more modern CT scan imaging, and PET scan staging.

There are few data regarding the tolerability of 5-FU and mitomycin chemoradiation in this elderly patient population. Published studies in other malignancies suggest benefit for treatment of elderly patients. A retrospective analysis of elderly colon cancer Medicare patients revealed only a modest increase in toxicity for those undergoing adjuvant chemotherapy (Schrag *et al*, 2001). Two European rectal cancer trials observed a benefit for elderly persons treated with 1 cycle of 5-FU and mitomycin with radiation (Valentini *et al*, 1997; Allal *et al*, 1999). All patients were over the age of 75. Neither trial reported grade 4 toxicity or any treatment-related death. Results observed were consistent with that reported for younger patients (Coia *et al*, 1991a, 1991b; Smith *et al*, 1998).

This regimen may be an appropriate alternative for oesophageal cancer patients who cannot tolerate standard therapy with cisplatin and 5-FU chemoradiation, and for older patients who have a heavy comorbidity burden and are deemed non-surgical candidates. In addition, two cycles of brief in-patient treatment weeks 1 and 5 allows for close patient monitoring. Recent studies have from our institution have also indicated that using non-5-FU containing chemotherapy regimens with concurrent radiotherapy may also have a potential to lower toxicity of combined chemoradiotherapy. In two phase I trials combining either weekly paclitaxel (Brenner *et al*, 2004) or weekly irinotecan (Ilson *et al*, 2003) with a weekly schedule of relative low-dose cisplatin and concurrent radiotherapy, gastrointestinal, and haematologic toxicities were relatively mild. These regimens also offer promise as an alternative to conventional 5-FU and cisplatin. Recent data also continue to support the use of 5-FU and mitomycin as a radiosensitising regimen in gastrointestinal cancers. A recent US Intergroup trial in anal cancer compared conventional 5-FU, mitomycin, and radiation to the use of induction chemotherapy with 5-FU and cisplatin, followed by combined 5-FU, cisplatin and radiation (Ajani *et al*, 2006). The 5-FU and cisplatin arm had an inferior outcome compared to conventional 5-FU and mitomycin, and the results underscore the use of this regimen as a treatment standard in anal cancer.

Older cancer patients may not be offered or may defer treatment secondary to the perception of increased toxicity. Our responses to treatment appear comparable to published chemoradiation studies observed in younger patients. Our investigation does not reveal increased haematologic toxicity from chemoradiation in this elderly cohort. Because of the retrospective nature of this analysis, non-haematologic toxicity data may be under-reported, as patients were not treated in the context of a prospective clinical trial. However, there was no need for placement of feeding tubes during chemoradiation treatment, although three patients required feeding tubes before initiation of therapy secondary to dysphagia for nutrition support.

Limitations regarding the generalisability of our results include the small cohort size, and the retrospective nature of this analysis resulting in significant patient selection for therapy tolerance. However, the majority of patients were over 70 years (92%), with locally advanced disease (84%) and more than 1 comorbid condition (84%). The median overall survival of 35 months observed in this elderly cohort suggest similar results to that observed in more toxic chemoradiation regimens in much younger oesophageal cancer patients. There is a need for clinical investigations evaluating the role of chemoradiation in elderly oesophageal cancer patients.

## Figures and Tables

**Table 1:** Patient demographics

Median age (range)	77 (66–88)
Median Karnofsky performance status:	80 (70–90)
Adenocarcinoma : squamous	12 : 13
	
*Stage*	
T4N1	2 (8%)
T3NX	5 (20%)
T3N1	10 (40%)
T3N0	4 (16%)
T2N0	4 (16%)
	
*Comorbidities*	
Diabetes	5 (20%)
Pulmonary disease	6 (24%)
Coronary artery or peripheral vascular	9 (36%)
	
*Disease*	
Median Charlson score	2
Charlson score 2 or more	18 (72%)

**Table 2:** Haematologic toxicity (*N*=25)

	**Mean**	**Grade 3**	**Grade 4**
Leukopaenia (k *μ*l^−1^)	2.5	3 (12%)	4 (16%)
Granulocytopaenia (k *μ*l^−1^)	1.4	6 (24%)	1 (4%)
Anemia (g dl^−1^)	10.6	0	1 (4%)
Thrombocytopaenia (k *μ*l^−1^)	110.0	0	2 (8%)
